# A novel likely pathogenic variant in the *FBXO32* gene associated with dilated cardiomyopathy according to whole‑exome sequencing

**DOI:** 10.1186/s12920-022-01388-5

**Published:** 2022-11-07

**Authors:** Serwa Ghasemi, Mohammad Mahdavi, Majid Maleki, Iman Salahshourifar, Samira Kalayinia

**Affiliations:** 1 Department of Biology, Science and Research Branch, Islamic Azad University, Tehran, Iran; 2 Rajaie Cardiovascular Medical and Research Center, Iran University of Medical Sciences, Tehran, Iran; 3 Cardiogenetic Research Center, Rajaie Cardiovascular Medical and Research Center, Iran University of Medical Sciences, Tehran, Iran

**Keywords:** Dilated cardiomyopathy, Genetic, *FBXO32*, Whole-exome sequencing, Variant

## Abstract

**Background:**

Familial dilated cardiomyopathy (DCM) is a genetic heart disorder characterized by progressive heart failure and sudden cardiac death. Over 250 genes have been reported in association with DCM; nonetheless, the genetic cause of most DCM patients has been unknown. The goal of the present study was to determine the genetic etiology of familial DCM in an Iranian family.

**Methods:**

Whole-exome sequencing was performed to identify the underlying variants in an Iranian consanguineous family with DCM. The presence of the candidate variant was confirmed and screened in available relatives by PCR and Sanger sequencing. The pathogenic effect of the candidate variant was assessed by bioinformatics analysis, homology modeling, and docking.

**Results:**

One novel likely pathogenic deletion, c.884_886del: p.Lys295del, in F-box only protein 32 (muscle-specific ubiquitin-E3 ligase protein; *FBXO32*) was identified. Based on bioinformatics and modeling analysis, c.884_886del was the most probable cause of DCM in the studied family.

**Conclusions:**

Our findings indicate that variants in *FBXO32* play a role in recessive DCM. Variants in *FBXO32* may disturb the degradation of target proteins in the ubiquitin–proteasome system and lead to severe DCM. We suggest considering this gene variants in patients with recessively inherited DCM.

## Introduction

Dilated cardiomyopathy (DCM) is a common cause of heart failure and is characterized by the dilatation and impaired function of 1 or both ventricles, resulting in an ejection fraction below 50% [1–3]. The incidence of DCM is 7 cases per 100,000 people yearly, with the disease affecting 1 in 250 individuals in the general population, worldwide [4, 5]. DCM may be considered the primary indication of heart transplantation [2] and can be developed by such agents as infections, toxins, drugs, nutritional deficiencies, genetic variants, inborn metabolism problems, endocrine disorders, immune and neuromuscular disorders, and the structure/function disruption of sarcomeres, cytoskeletons, sarcoplasmic reticula, and nuclear envelopes [1, 6, 7]. DCM is genetically heterogeneous, and approximately 30–48% of all cases are genetic forms. To date, over 250 genes have been reported in association with DCM [8]. 90% of DCM cases have autosomal dominant inheritance, and about 10% are X-linked, autosomal recessive, or mitochondrial [5]. F-box only protein 32 (muscle-specific ubiquitin-E3 ligase protein; *FBXO32*) maps to chromosome 8q24.13, and its alternative splicing leads to 3 transcripts: NM_148177, NM_001242463, and NM_058229. The latter transcript is the largest one in that contains 9 exons and 6744-bp nucleotides. The *FBXO32* gene encodes the muscle-specific ubiquitin-E3 ligase protein with a length of 355 amino acids, also known as “atrogin-1/MAFbx”, in skeletal muscles and cardiomyocytes [9]. This protein localizes at the sarcomere and plays a critical role in muscle atrophy, cardiac hypertrophy, and atrophy development [10]. The ubiquitin-E3 ligase protein acts in the SCF complex (Skp1, Cdc53/Cullin1, and the F-box protein), causing ubiquitin-dependent proteolysis in the cells [11]. The first homozygous missense variant in *FBXO32*, c.727G > C: p.Gly243Arg, was discovered by Al-Yacoub et al. [10], who reported that the variant destroyed the SCF complex and dysregulated the autophagy/lysosomal system in early-onset DCM.

Next-generation sequencing (NGS) provides a cost-effective and accurate diagnostic method to identify variants in patients with DCM [12–14]. In the present study, via whole-exome sequencing (WES), we detected a novel deletion, c.884_886del: p.Lys295del, in *FBXO32* that may play a role in DCM pathogenesis. In addition, we conducted a comprehensive review of all reported *FBXO32* variants in patients with DCM. To our knowledge, the present study is the first report of the inframe single amino acid deletion causing DCM in homozygosity the world over.

## Methods

### Family recruitment and clinical evaluation

**Fig. 1:**
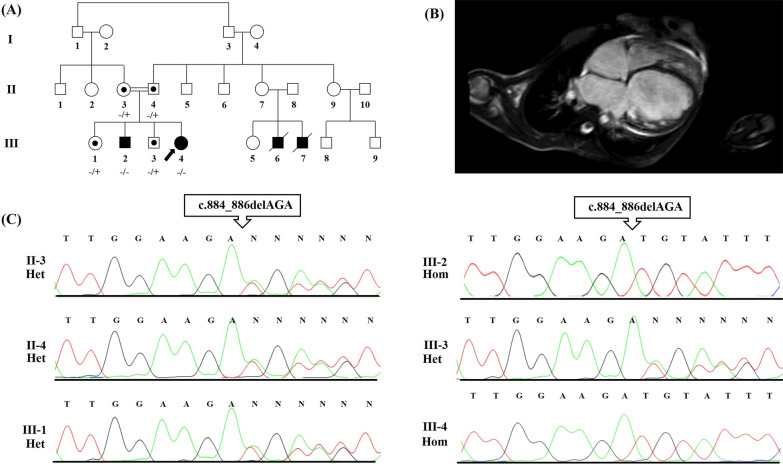
The image presents the pedigree of the family with dilated cardiomyopathy (DCM), as well as the results of magnetic resonance imaging (MRI) and the sequencing chromatograms of the mutated nucleotide in the *FBXO32* gene. **A** The pedigree of the family with DCM is presented herein. The proband (indicated by the arrow) was a candidate for heart transplantation. **B** The MRI of the proband (III-4) is presented herein. **C** The Sanger sequencing results of the *FBXO32* gene in the patient and her family members are shown here. The patients carried a homozygous missense variant: c.884_886del: p.Lys295del

An Iranian family comprising parents and 4 children was referred to the Cardiogenetics Research Center, Rajaie Cardiovascular Medical and Research Center, Tehran, Iran, for genetic diagnostic testing (Fig. 1A). The parents who were consanguineous, had no DCM. The father was 53 years of age, and the mother was 48 (Fig. 1A, II-3, II-4). Two children suffered from DCM (Fig. 1A, III-2, III-4), and the 2 others were healthy (Fig. 1A, III-1, III-3). The index patient (Fig. 1A, III-4) was a 25-year-old girl first referred to our center aged 18 for chest pain and palpitations. Her medical history was unremarkable as it featured no syndromic features such as metabolic disorders, premature aging, and skeletal muscle diseases. Her older brother exhibited the same DCM symptoms. Interestingly, the pedigree had 2 now-deceased family members with DCM (Fig. 1A, III-7, III-8). Routine cardiovascular examinations, including magnetic resonance imaging (MRI), thorough physical examinations, and electrocardiography (ECG), were performed. The MRI results determined DCM (Fig. 1B), but the electrocardiography results were normal.

The current study was conducted following the Declaration of Helsinki and was approved by the Ethics Committee of Rajaie Cardiovascular Medical and Research Center (IR.RHC.REC.1399.019). Informed consent to participate in the study was obtained from all subjects.

### DNA extraction, WES, and in silico analysis

Peripheral blood samples were collected from all the family members. Genomic DNA was extracted using the DNSol Midi Kit (Roche: Product No. 50072012). In the first step of the genetic investigation, the hotspots of 5 common genes—*TTN*, *MYH7*, *MYBPC3*, *BAG3*, and *LMNA*—were screened by polymerase chain reaction (PCR) and Sanger Sequencing, which revealed no pathogenic variant. Further surveys were carried out via WES on the proband (Fig. 1A, III-4) at Macrogen (Seoul, South Korea). All the exomes were enriched and captured using the SureSelect XT Library Prep Kit. Sequencing was carried out on an Illumina HiSeq 4000 platform following the manufacturer’s protocol of Illumina, and paired‐end reads were produced. A depth of greater than 7 and a read quality value (Phred Score) of greater than 20 were considered for the next steps. The quality of the sequencing reads was controlled using FastQC. Low-quality reads were trimmed; subsequently, the Burrows–Wheeler Aligner (BWA-MEM v.07.17) [15] was used to align the clean reads to the reference genome (UCSC Genome Browser, hg19). Single-nucleotide polymorphism and insertion and/or deletion calling was performed by the Genome Analysis Toolkit (GATK, v.4.1.4.1) [16]. All the determined variants were annotated by ANNOVAR [17] and filtered according to the 1000 Genomes Project, the Exome Aggregation Consortium (ExAC), and Genome Aggregation Database (gnomAD) and an Exome Sequencing Project ESP6500 minor allele frequency (MAF) of 0.005. The candidate variants were analyzed with bioinformatics tools, consisting of CADD (https://cadd.gs.washington.edu/), SIFT (https://sift.bii.a-star.edu.sg), PolyPhen-2 (https://genetics.bwh.harvard.edu/pph2), MutationTaster (www.mutationtaster.org), and PROVEAN (https://provean.jcvi.org), according to the 2015 guidelines of the American College of Medical Genetics and Genomics (ACMG) [18]. In addition, the regions of the variants were evaluated concerning conservation using the GERP++ score. The conservation analysis of the protein change position was performed by comparing the amino acid sequences of different species on the CLUSTALW Web Server (https://www.genome.jp/tools-bin/clustalw).

### Confirmation and segregation analysis

Segregation analysis was conducted via PCR-direct Sanger sequencing to evaluate the family members. For variant amplification, 2 primer pairs were designed using the Gene Runner v.6.0 software with the following sequences: forward: 5′-AGGGAAGGATAGGCATTGTTG-3′ and reverse: 5′-GCTGGCACTCTTGGCTCT-3′. PCR was performed on a SimpliAmp Thermal Cycler (Thermo Fisher Scientific) with 100 ng DNA, 1.5 mmol/L of MgCl2, 200 mmol/L of dNTP, 10 pmol/L of the primers, and 1 U of Taq DNA polymerase (Amplicon, UK). Then, incubation at 95 °C for 5 min and 35 amplification cycles (30 s at 95 °C, 30 s at 62 °C, and 30 s at 72 °C) was applied. The PCR products were sequenced on an ABI Sequencer 3500XL PE (Applied Biosystems) using the same primer sets, and the sequences were analyzed using FinchTV 1.4.0 (Fig. 1C).

### Computational modeling

#### FBXO32 and importin α3

**Fig. 2:**
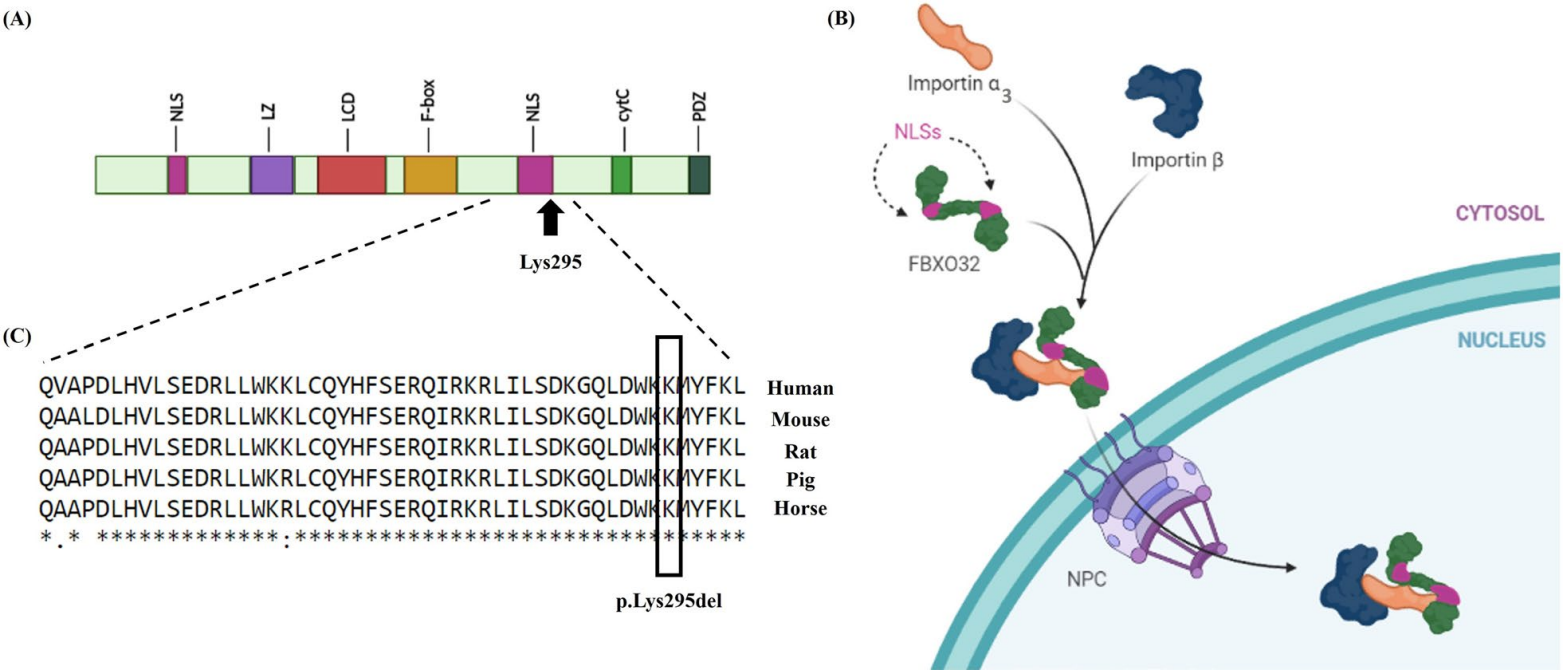
The image depicts the potential mechanism of FBXO32, as well as the location and conservation of the mutated amino acid. **A** The different domains of FBXO32 are illustrated here. The image presents a schematic representation of the FBXO32 structure, containing the leucine zipper domain, the leucine-charged residue-rich domain (LCD), nuclear export signal (NES-like motif) in the LCD domain, 2 highly conserved nuclear localization signals (NLS), the F-Box domain (F-Box), the PDZ domain (PDZ), and the Cytochrome c-like domain (CytC). **B** The mechanism of the importin α3/β-mediated nuclear import of FBXO32 is depicted here. The NLS domain is bound to importin α3; then, importin β binds to importin α3 for transmission through the nuclear pore complex (NPC). **C** The alignment of conserved NLS residues from different FBXO32 orthologs was compared using the CLUSTALW Web Server. The lysine amino acids are shown in the box

FBXO32 (MAFbx1/Atrogin1) is a muscle and cardiomyocyte-specific F-Box protein that is a component of the SCF complex [9, 10]. The different domains of the FBXO32 consist of the leucine zipper domain [19], the leucine-charged residue-rich domain (LCD), the nuclear export signal (NES-like motif) in the LCD domain, 2 highly conserved nuclear localization signals (NLS), the F-Box domain (F-Box), the PDZ domain (PDZ) and the cytochrome c-like domain (CytC) [10] (Fig. 2A). The F-Box domain binds directly to the Skp1 protein in the SCF complex, which leads to the binding of carboxy-terminal domain-specific substrates [20]. The variants in the F-Box domain disrupt the SCF assembly autophagy [21]. The presence of both NLS and LCD domains is necessary for the nuclear localization of FBXO32 [22]. The proteins are exchanged between the nucleus and the cytoplasm through the nuclear pore complex (NPC), located in the nuclear envelope. Transmission through the NPC is mediated by 2 essential components—importin α3 and importin β1—and NLS sequences in the transferable protein. Further, human genomes encode 7 subtypes of importin α: α1, α3, α4, α5, α6, α7, and α8.

Based on AceView (https://www.ncbi.nlm.nih.gov/IEB/Research/Acembly/), of all the subtypes, importin α3 has the highest expression in the heart. For the transition of the FBXO32 protein, first, the NLS sequence is recognized by the importin α3 adaptor molecule, and then importin β1 binds to importin α3 to form a ternary complex called “the nuclear pore-targeting complex (PTAC)”. This complex is translocated by the NPC via importin β1 activity before it is transferred into the nucleus [23] (Fig. 2B).

#### Homology modeling and docking

Computer-assisted molecular docking was performed to investigate the binding between importin α3 and FBXO32 (normal and the Lys295del variant) on the HADDOCK Web Server (https://wenmr.science.uu.nl/haddock2.4/) [24, 25]. In the first step, the 3D structure of the human importin α3 was downloaded from the Protein Data Bank (PDB, https://www.rcsb.org/) (ID: 6BW9, resolution: 1.6 Å). Since PDB lacks the 3D structure of FBXO32, the SWISS-MODEL Server (https://swissmodel.expasy.org/) was used to obtain the 3D structure of the human FBXO32 (normal and the Lys295del variant) [26–30]. All heteroatoms, including water molecules, ions, and native ligands, were removed from the parent structures, and polar hydrogens were added using ViewerLite v.5. For the docking study, the energy was minimized with the aid of the YASARA Energy Minimization Server (http://www.yasara.org/minimizationserver.htm) [31]. The 3D structures of the compounds, wild-type/Lys295del FBXO32-importin α3, were imported as files into the YASARA View v.20.12.24 to be saved as PDB files. After energy minimization and excess heteroatom removal, the binding sites were predicted using the Computed Atlas of Surface Topography of proteins (CASTp) Server (http://sts.bioe.uic.edu/) [32]. The HADDOCK Web Server was utilized for molecular docking studies. Eventually, the top-ranked pose, as judged by the docking score, was subjected both to visual analysis using PyMOL v.2.5.2 and to molecular interaction viewing using LigPlus+ v.2.2.4 [33, 34].

## Results

### Molecular findings

WES was performed on the proband (Fig. 1A, III-4) to discover the causative variant. A novel likely pathogenic variant, c.884_886del, was found in the eighth exon of the *FBXO32* gene. This variant leads to delete lysine amino acid at site 295. Notably, the variant has not yet been reported in the 1000 Genomes Project, ExAc, gnomAD, HGMD, and ClinVar or publications. According to the ACMG, c.884_886del was determined as a likely pathogenic variant (criteria: PM2, PM4, PP1, PP3, and PP5). This nonsense variant was considered as a damaging variant by CADD, SIFT, PolyPhen-2, PROVEAN, FATHMM, and GERP++. The variant was confirmed in the proband (Fig. 1A, III-4) by PCR and Sanger sequencing in the homozygous state. It was also detected in the proband’s affected brother (Fig. 1A, III-2) as a homozygote. The other members of the family—the mother, the father, the sister, and the brother (Fig. 1A, II-3, II-4, III-1, III-3), respectively)—had this variant in a heterozygous state (Fig. 1C). DNA from the other pedigree members was not available. In addition, based on CLUSTALW results, Lys295 was located in the conserved part of the FBXO32 protein (Fig. 2C).

### Three‑dimensional protein structure modeling

**Fig. 3:**
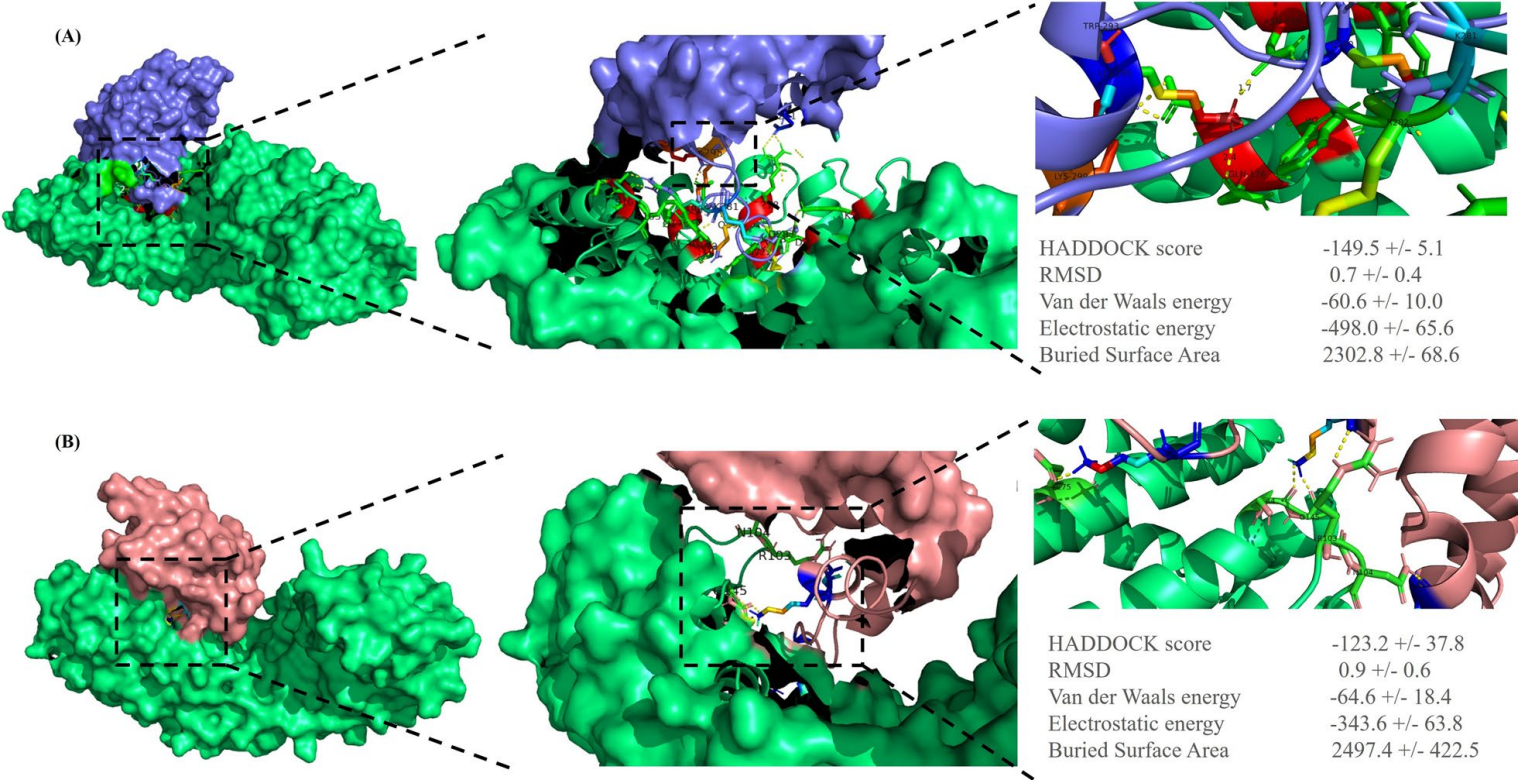
The image presents the top complex structures for normal and mutant FBXO32 in interaction with importin α3 (PDB ID: 6BW9), as well as their HADDOCK scores and related energies. **A** The binding site of wild-type FBXO32 is surrounded by 8 amino acids including Lys288, Gln290, Lys295, Lys299, Arg282, Lys281, Glu261, and Asp287 that binds to importin α3. The panel shows the 3D strong interaction between importin α3 (green) and the wild-type FBXO32 (blue). **B** The panel shows the 3D weak interaction between importin α3 (green) and the mutant FBXO32 (pink). The binding site of Lys295del FBXO32 is surrounded by 4 amino acids including Lys294, Lys288, Val258, and Asp292 that binds to importin α3. The images were obtained by using PyMOL v.2.5.2. (The yellow dashed lines represent the hydrogen bonds)

**Fig. 4:**
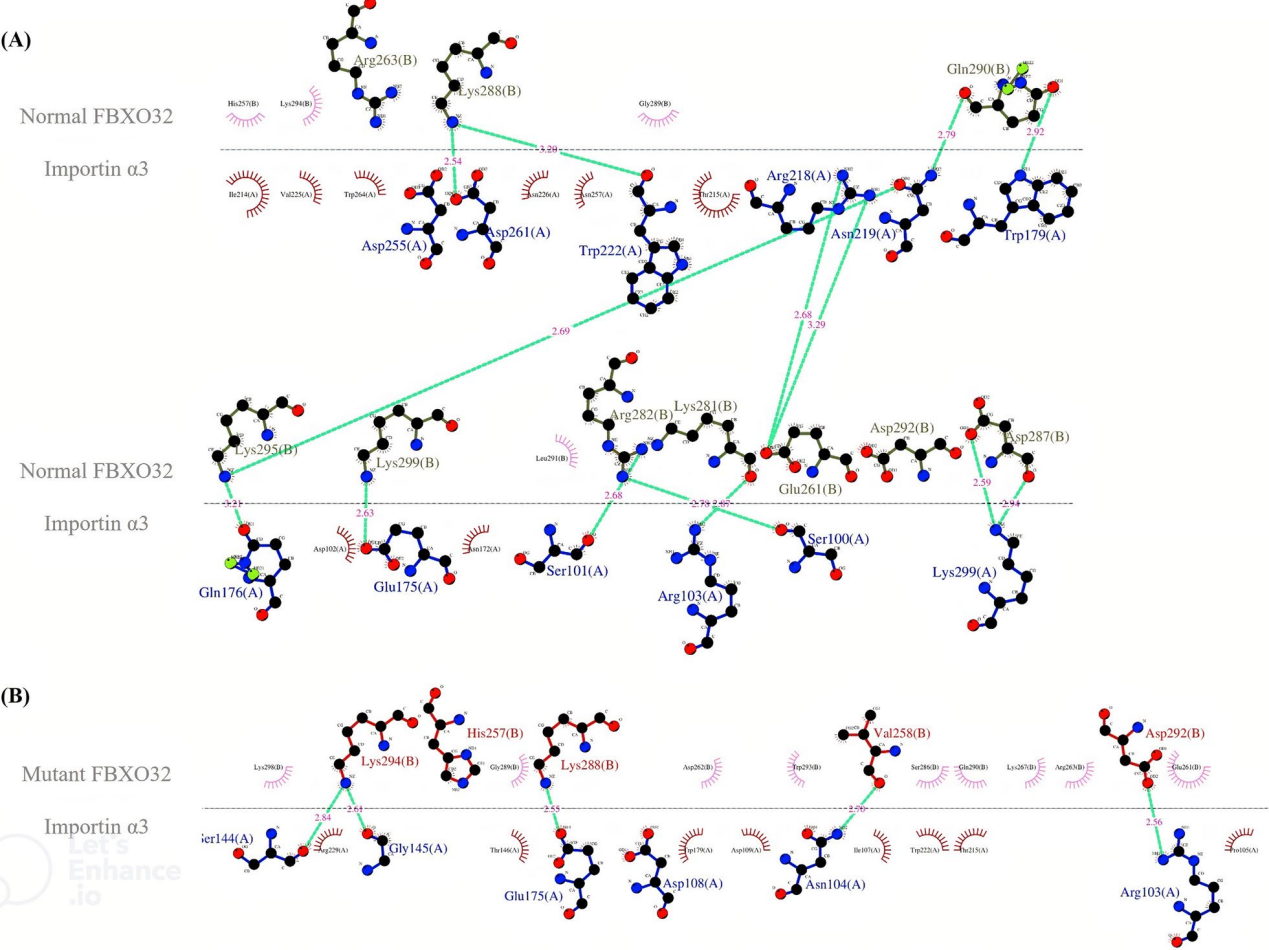
The image demonstrates the molecular interactions between the normal (**A**) and mutant (**B**) FBXO32 and importin α3 generated by LigPlus+ v.2.2.4. (The dotted green line indicates hydrogen bonding, with bond distance). In the wild-type FBXO32, 8 amino acids (Lys288, Gln290, Lys295, Lys299, Arg282, Lys281, Glu261, and Asp287) formed hydrogen bonds with 11 amino acids importin α3 (Asp261, Trp222, Arg218, Asn219, Trp179, Gln176, Glu1754, Ser101, Arg103, Ser100, and Lys299), whereas in the Lys295del FBXO32, just four 4 amino acids (Lys294, Lys288, Val258, and Asp292) formed hydrogen bonds with 5 amino acids importin α3 (Ser144, Gly145, Glu175, Asn104, and Arg103)

The FBXO32 p.Lys295del variant is located in the NLS domain of the protein and may prevent the binding of importin α3 and FBXO32, disturbing the entry of FBXO32 into the nucleus. The results of the molecular docking of the compounds indicated a change in the interaction between the human wild-type/Lys295del FBXO32 and importin α3. Among the clusters resulting from the docking experiments, the cluster with the lowest Z scores was the best. The docking scores of the Lys295del variant and the wild-type FBXO32 with importin α3 were − 123.2 ± 37.8 kcal/mol and − 149.5 ± 5.1 kcal/mol, respectively. In addition, the position of importin α3 in the interaction with the Lys295del FBXO32 showed a change in comparison with the wild-type FBXO32. The interaction analysis showed that the binding site of the wild-type FBXO32 was surrounded by 8 amino acids—Lys288, Gln290, Lys295, Lys299, Arg282, Lys281, Glu261, and Asp287, which formed 14 hydrogen bonds with importin α3 (Fig. 3A), whereas in the Lys295del FBXO32, just 4 amino acids—Lys294, Lys288, Val258, and Asp292—formed 5 hydrogen bonds with importin α3 (Fig. 3B). Indeed, Lys295 in the wild-type FBXO32 formed 2 hydrogen bonds with importin α3, and the lack of this amino acid in the Lys295del FBXO32 reduced the hydrogen bond and affinity with importin α3. Moreover, this variant led to fewer hydrogen bonds at the surface of the FBXO32-importin α3 complex, causing lower binding affinity (Fig. 4).

## Discussion

DCM, as a primary cause of heart failure, is a very complex disease genetically [1]. Although the molecular basis of DCM is unclear, WES has emerged as a powerful and cost-effective method capable of filling this gap. The first report of the *FBXO32* variant described a Saudi family with DCM in 2015, according to which the variant was located in the F-box domain of Atrogin-1 and changed glycine 243 to arginine [10]. In the present study, conducted on an Iranian family with members suffering from DCM, we identified a novel likely pathogenic variant, c.884_886del, in *FBXO32* that may be associated with DCM pathogenesis. This variant led to the deletion of lysine in the highly conserved NLS domain of the Atrogin-1 protein. Al-Hassnan et al. [9] demonstrated substitution of a highly conserved amino acid had functional consequences, severely impairing binding to SCF proteins. The results of a study by Julie et al. [22] demonstrated that the deletion of either NLS1 or NLS2 induced the localization of FBXO32 to the cytosol, the absent transition of FBXO32 to the nucleus, and the disruption of SCF complex formation in the nucleus. The modeling result of our study provided evidence that the *FBXO32* c.884_886del variant compromised the formation of the SCF complex in the nucleus, impaired autophagy, and increased apoptosis.

The ubiquitin–proteasome system is responsible for the destruction of misfolded proteins in the cell. Further, it works in both the nucleus and the cytoplasm via 2 main pathways: autophagy and proteasomal pathways [35]. The SCF complex, a core member of the ubiquitin–proteasome system, is responsible for the degradation of native and misfolded proteins. Variants of *FBXO32* in the SCF complex decrease the activation of the unfolded protein response and increase the expression of transcription factor C/EBP homologous protein (CHOP). Indeed, these variants increase CHOP-associated apoptosis through the mitochondria-dependent pathway, which is followed by damage to the autophagy/lysosomal system [36]. FBXO32 regulates the half-life of charged multivesicular body protein 2B (CHMP2B), an important mediator in autophagosome-lysosome fusion [36]. Al-Yacoub et al. [10] evaluated patients with DCM and reported a decline in CHMP2B expression, resulting in defective autophagy. In another study, *FBXO32*-knockout mice showed intracellular protein accumulation and cardiomyocyte apoptosis, causing cardiomyopathy and premature death via impaired autophagy [21]. Our docking analysis revealed that the p.Lys295del variant altered the formation and function of the SCF complex in the nucleus. Our protein–protein docking analysis suggested that p.Lys295del could lead to fewer hydrogen bonds and hydrophobic interactions at the surface of FBXO32 and importin α3, decreasing their binding affinity and lessening the likelihood of the successful transfer of FBXO32 to the nucleus. Nevertheless, additional functional studies are needed to analyze the pathomechanisms underlying FBXO32-associated DCM.

In conclusion, our study contributes to the genetic diagnosis of families with DCM and suggests that FBXO32 variants could play a role in recessive DCM [10]. WES is a powerful tool to identify disease-causing variants in complex diseases that have hitherto remained unknown.

## Accession number

The accession number of the variant in ClinVar is as follows:

NM_058229.4 (FBXO32): c.884_886del (p.Lys295del): VCV001188824.1

## Data Availability

All data generated or analyzed during this study are included in this published article.
